# The HeartHealth Program: A Mixed Methods Study of a Community-Based Text Messaging Support Program for Patients With Cardiovascular Disease From 2020 to 2024

**DOI:** 10.2196/68896

**Published:** 2026-03-11

**Authors:** Brodie Sheahen, Liliana Laranjo, Ritu Trivedi, Tim Shaw, Gopal Sivagangabalan, James Chong, Aravinda Thiagalingam, Sarah Zaman, Pierre Qian, Anupama Balasuriya Indrawansa, Clara Kayei Chow

**Affiliations:** 1 Westmead Applied Research Centre, The University of Sydney, Level 6, Block K, Entrance 10, Westmead Hospital, Hawkesbury Rd, Sydney, 2145, Australia, 61 0488975865; 2 School of Health Sciences, The University of Sydney, Sydney, Australia; 3 Charles Perkins Centre, The University of Sydney, Sydney, New South Wales, Australia; 4 Department of Cardiology, Westmead Hospital, Sydney, Australia

**Keywords:** text messaging, Short Message Service, SMS, secondary prevention, implementation evaluation, cardiovascular disease, digital health intervention

## Abstract

**Background:**

The HeartHealth program is a 6-month SMS text messaging–based support program offered to patients with a recent cardiovascular hospitalization or recent cardiovascular clinic visit in Western Sydney, Australia. Its customized content focuses on cardiovascular risk factors, lifestyle, treatments, and general heart health information.

**Objective:**

This study aimed to evaluate the implementation of the HeartHealth program.

**Methods:**

A mixed methods study was conducted assessing program reach, effectiveness, implementation, and maintenance using program data, participant feedback surveys, and staff focus group discussions. Consecutive adult patients who had attended cardiology clinics or had been discharged from cardiology hospitalization at Westmead Hospital, between April 2020 and April 2024, were included in the analysis. Content analysis was used to interpret the qualitative data.

**Results:**

A total of 23,095 patients were invited, 8804 (38.1%) enrolled into the program, and 7964 out of 8804 (90.5%) completed the 6-month duration. Participants enrolled in the HeartHealth program had a mean age of 58.6 years, 60.3% (5302/8788) were male, and 62.4% (5382/8624) were recruited from an outpatient clinic setting. A total of 851,058 SMS text messages were sent, with 99.41% (846,009/851,058) delivered successfully. A total of 3533 out of 7964 (44.4% of program completers) participants completed the postintervention survey, and 4 HeartHealth staff members participated in a focus group discussion. Among the participants who completed the survey, 60.5% (2137/3533) reported that the program improved the healthiness of their diet, 53.6% (1894/3533) reported improved physical activity levels, and 56.1% (1982/3533) reported that it helped remind them to take their medications. Content analysis of participant feedback identified that the program was effective in prompting participants to change their diet, providing emotional support, reminding them of the importance of behavior change, improving their confidence in managing their health, and keeping participants focused. Key barriers included limited personalization, language options, and SMS text messaging scheduling flexibility. Recommended adaptations focused on enhancing personalization, greater engagement by local clinical teams, and expanding program dissemination.

**Conclusions:**

The program had a broad reach, translated to improved patient-reported health behaviors, and provided participants with needed support at low cost and low resource requirements. This analysis highlights the successful implementation and scalability of the HeartHealth program and provides key learnings for health systems that are looking to implement similar programs in the future.

## Introduction

Patient education is an important element of care of patients with chronic disease, and several international agencies now have a policy focus on improving patient education to support self-management [1-3]. Smoking cessation, physical activity, diet modification, weight management, and medication management are all important in cardiovascular disease (CVD) prevention, but current models of care are limited in support for patients to address these behaviors [4]. Most current health care models’ education and support are typically delivered through in-person consultation and group activities; however, this modality poses multiple barriers such as many travel requirements [5], cost [6], time, and lack of prioritization [7]. In addition, many health care services lack the resources and funding to deliver these, and programs have limited ability to cater to population diversity [8,9].

Digital health technologies present a scalable means to deliver customized patient education [10]. Several small- to medium-sized randomized controlled trials have shown mobile health texting interventions to improve patient CVD risk factors, including: low-density lipoprotein cholesterol levels [11], BMI [11], blood pressure [12], weight management [13], and smoking cessation [14]. Furthermore, these interventions report high rates of patient satisfaction [15] and usability [11]. Yet, despite the research to date, there are limited examples of real-world implementation and evaluation of large-scale digital education and support programs for cardiovascular and other chronic diseases.

In April 2020, the HeartHealth program was initiated and offered to patients with CVD discharged from cardiovascular services or clinics in Western Sydney, New South Wales. The program provides personalized cardiovascular education and support via SMS text messaging through a digital customization platform over a 6-month period and an opportunity to ask questions. This study aimed to evaluate the implementation of the HeartHealth program.

## Methods

### HeartHealth Program Description

The HeartHealth program was designed to reduce cardiovascular risk in patients with CVD or at high CVD risk. The HeartHealth program involved the delivery of regular semipersonalized cardiovascular education and support via SMS text message for 6 months. SMS text messages were sent approximately 3‐4 times per week. The message bank was developed by clinicians, academics, and patients and covered the following 5 modules: smoking, diet, physical activity, COVID-19, and general cardiovascular health. Messages were written to provide advice, education, motivation, and reminders aimed at improving cardiovascular risk factors and healthy lifestyle behaviors. SMS text messages would often be supplemented with a URL link to a website to enable access to further information on the message content. Participants were able to opt out of the program at any time through alerting staff through responding to the SMS text messages. The core structure of the program content was curated based on the previously published TextMe and TextMe2 programs [11,16]. Message content development was based on a range of theoretical frameworks spanning 3 phases of development as previously described [17,18], with program content actively reviewed and updated.

At registration, participants completed a survey detailing their baseline characteristics (hypertension, diabetes mellitus, hypercholesterolemia, smoking status, and diet preference). Algorithms selected messages from the message bank based on participants’ baseline characteristics, tailoring each program accordingly. Messages addressed the participants by their preferred name and provided the source of information; examples of SMS text messages have previously been outlined in program development protocols [17,18]; furthermore, Multimedia Appendix 1 provides examples of messages.

To disseminate SMS text messaging support programs at scale, our team at Westmead Applied Research Centre built a cloud-based digital platform “TextCARE” that can deliver multiple programs according to varying clinical algorithms, simultaneously. Hence, this enabled delivery of customized content to thousands of people concurrently. The HeartHealth program that started at Westmead Hospital in April 2020 continues to be deployed.

### Enrollment and Eligibility

Patients were identified either following attendance at a Westmead Hospital outpatient rapid access cardiology clinic or following discharge from an inpatient cardiology admission at Westmead Hospital. Patients were eligible for HeartHealth if they were aged 18 years or older, post hospital discharge from a cardiology admission, or had recently attended an outpatient cardiology clinic. Initially, the program was designed where consecutive eligible patients from the previous week were sent a single SMS text message to enroll into the HeartHealth program. Recruitment protocols were adapted in October 2021 so that participants who did not respond to the initial SMS text messaging invitation were followed up with a phone call from a HeartHealth staff member and offered program enrollment. Consent was obtained electronically, disseminated by an SMS text message, and captured on REDCap (Research Electronic Data Capture; Vanderbilt University).

### Ethical Considerations

This study was approved by the Western Sydney Local Health District Human Research Ethics Committee (approval number 2020/ETH01649). Consent for data collection was provided by participants at the time of program enrollment. All data collected are deidentified. There was no compensation provided to participants for their involvement with this study.

### Study Design

This study is a retrospective observational study that evaluated the implementation of the HeartHealth program, an existing program implemented as standard of care in the Western Sydney Local Health District, New South Wales, Australia. A mixed methods design assessing the HeartHealth program “Reach,” “Effectiveness,” “Implementation,” and “Maintenance” was used. Typically, an implementation evaluation would also assess program site “adoption”; however, as the HeartHealth program was intended to be rolled out only at one site, this component was not assessed. Three sources of data were collected: postintervention surveys, focus group discussions with organization staff, and program-related data. Data sources used for analysis are outlined in Table 1.

**Table 1.** Descriptions of the implementation evaluation domains including the domain definition, outcome measures, and data sources.

Domain	Definition	Outcome measures	Data sources
Reach	Reach was defined as the characteristics and proportion of patients who agreed to opt into the HeartHealth program following hospital discharge or outpatient clinic visit
		Participant enrollment	Program data^a^
Characteristics of patients who did and did not opt into the program	Program data Electronic medical health records (adapted with permission from Sheahen et al., [19])
Reasons patients did not opt into the program	Program data
Effectiveness	Effectiveness was defined as clinical improvements following the program delivery and was assessed by participant behavior and knowledge changes
		Participant lifestyle behavior changes	Postintervention survey
Participant health knowledge and behavior.	Postintervention survey
Implementation and maintenance	Implementation and maintenance were defined as the extent the staff members implemented and maintained the program as intended as well as the participant’s perception of appropriate program delivery
		Program fidelity, attrition, and organization requirements	Program data
Program resources and costs	Program data
Participant SMS text message interaction	Program data
Program barriers (organization and individual levels)	Focus group discussion Postintervention survey
Program enablers (organization and individual levels)	Focus group discussion Postintervention survey
Program adaptations required for long-term maintenance (organization and individual levels)	Postintervention survey Focus group discussion
Program adaptations made by HeartHealth staff	Focus group discussion Program data

a Data obtained from participants and staff members during enrollment, throughout the program, and at program completion as per the programs standard practice.

### Data Collection

#### Postintervention Surveys

All participants in the HeartHealth program were invited to complete an assessment survey at the end of the intervention (Multimedia Appendix 2). The survey was designed by The University of Sydney staff to evaluate the program, asking participants for feedback on the program’s impact, such as changes in lifestyle behaviors, what they enjoyed, and what could be improved. The survey was distributed via REDCap.

#### Focus Group Discussion

All current HeartHealth staff members (n=4) involved with the operationalization of the HeartHealth program were invited to partake in a single focus group discussion regarding the reach, effectiveness, implementation, and maintenance of the HeartHealth program (Multimedia Appendix 3).

#### HeartHealth Program Data

From the onset of the HeartHealth program, staff members recorded and stored data on participant outreach, enrollment, opt-out, and responses to SMS text messages. On a weekly basis, staff members would record, categorize, and store the program data, enabling thorough and complete analysis on the reach and implementation components of our analysis. These data were stored securely on The University of Sydney Research Data Store platform.

#### Data or Statistical Analysis

Statistical analysis was undertaken using R statistical software (version 4.2.0; R Core Team). Categorical data, including quantitative survey data, program attrition data, and participant demographic data, are presented as frequencies and percentages. Qualitative data assessing participant and staff perspectives of program barriers, enablers, areas of required adaptations, interaction with SMS text messages, and implementation of the program are analyzed via content analysis [20]. One researcher (BS) familiarized themself with the data and inductively coded the data into themes and subthemes. Three researchers (BS, RT, and LL) then reviewed and discussed established themes and subthemes, which were repeatedly adapted until agreement was reached on final theme and subtheme formation. HeartHealth participants were excluded from the “Effectiveness” section of the analysis if they did not complete the entire 6-month program duration.

## Results

### Reach and Participant Enrollment

**Figure 1.**
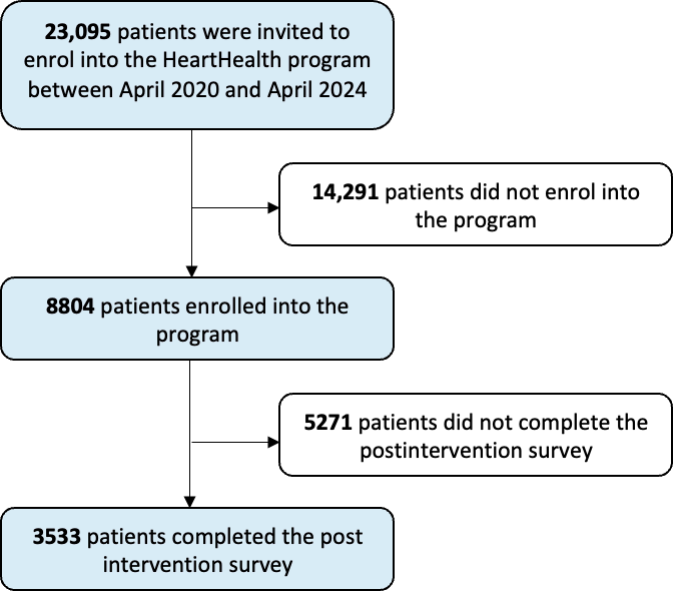
HeartHealth program enrollment schema outlining the number of patients invited, enrolled, and completed the postintervention survey.

A total of 23,095 patients, who had either attended a Westmead Hospital cardiology clinic or were discharged from Westmead Hospital cardiology unit between April 2020 and April 2024, were offered the HeartHealth program. A total of 14,291 patients did not opt into the program, and 8804 patients consented to participate in the program (enrollment rate: 8804/23,095, 38.1%; Figure 1).

Most patients were invited to enroll in the program following an outpatient clinic review (59.7% vs 40.3%); consequently, the HeartHealth program cohort primarily consisted of patients recruited from a clinic setting. HeartHealth participants were slightly younger (58.6 years vs 61.7 years) than the nonenrollee patients; however, gender distribution was similar (Table 2). Extracted electronic medical record data on comorbidities were obtained for 4324 HeartHealth participants and 7218 nonenrollee patients from April 2020 to April 2022. During this period, program participants were more likely to report English as their preferred language than nonenrollee patients (82.1% vs 76.0%, respectively) and less likely to have prior CVDs than nonenrollee patients (eg, ischemic heart disease 15.2% vs 20% and heart failure 25.4% vs 32.7%, respectively; Multimedia Appendix 4—adapted with permission from Sheahen et al [19]).

**Table 2.** Characteristics of participants who opted into the HeartHealth program and those who did not opt in (nonenrollee patients), including age, gender, and site of recruitment.

Characteristics	HeartHealth participants (n=8804)	Nonenrollee patients (n=14,291)	Overall (N=23,095)
Age (years), mean (SD)	58.6 (16.1)	61.7 (17.8)	60.5 (17.2)
Missing, n	12	10	22
Sex			
Male, n/N (%)	5302/8788 (60.3)	8647/14,263 (60.6)	13,949/23,051 (60.5)
Female, n/N (%)	3486/8788 (39.7)	5616/14,263 (39.4)	9102/23,501 (39.5)
Missing, n	16	28	44
Site of recruitment			
Hospital setting, n/N (%)	3242/8624 (37.6)	5872/14,013 (41.9)	9114/22,637 (40.3)
Clinic setting, n/N (%)	5382/8624 (62.4)	8141/14,013 (58.1)	13,523/22,637 (59.7)
Missing, n	180	278	458

### Effectiveness

A total of 3533 out of 7964 (44.4% of participants who completed the program) participants completed the postintervention survey. Of those participants who completed the survey, most participants reported that the program helped them improve the healthiness of their diet (n=2137, 60.5%), increase their exercise levels (n=1894, 53.6%), and remind them to take their medications (n=1982, 56.1%) [1]. Content analysis of participant feedback identified that HeartHealth participants reported that the program was effective in improving their overall health (Textbox 1). The first theme highlighted that the program translated into improved health behaviors through initiating lifestyle changes, empowering patients to engage in self-management of their health, and reinforcing healthy behaviors. The second theme demonstrated that the program improved patients’ well-being and psychological health through improving patients’ sense of care, accountability, motivation, and positivity.

Textbox 1. **Themes and subthemes on participant benefits of the HeartHealth program.**

Theme 1: Program translated to effective health behavior change

**Initiating lifestyle change**

- “Extremely useful [HeartHealth program], I’ve made changes to my lifestyle and feel I’m taking more control over myself.”
- “Thanks to your support I am now on the right path to getting better, I first thought I wouldn’t make next year . By slowly following the beginning of program and taking it slowly day by day . I didn’t have any blockages in my heart, but my [myocardial infarction] was caused through either trauma or stress, so I naturally sourced methods from your program to help me.”
- “The program has prompted me to reduce my salt intake. While I already knew that salt should be reduced in our diets, it was useful being prompted about it and having suggestions on how to reduce salt intake.”
- “I was overweight, I didn’t eat [many] vegetables, but now I do. I didn’t walk much, now I walk every day . This program has changed my [lifestyle].”
- “I was pleased to be part of the trial which was a big factor in me losing 20 KG and becoming fitter.”
- “The only unhealthy thing about my lifestyle is smoking cigarettes, those text reminders did make me think about quitting though – so I guess that’s a positive.”
- “The smoking messages encouraged me to quit smoking. 3 months [since I] quit.”

**Empowering people to engage in self-management**

- “I found all the messages were very useful for me as it empowered me to do more activities, eat healthy, take my tablets and to stay healthy. I’m well and improved a lot beyond expectation. Thank you so much.”
- “After following this program I am confident I can follow through with the activities suggested and have healthy foods in the future for improvement to my physical health and internal health.”
- “Most of the messages are useful and doable. I have gained a lot of important information necessary to maintain a healthy lifestyle.”

**Reinforcement of healthy habits**

- “Short and sharp reminders helped me re-focus especially with regard to diet and exercise.”
- “Good work. I’m glad I’m doing this program. Keeps me more conscious of my lifestyle and what needs improvement for a longer and healthier life.”
- “The messages keep me focused, and I review them weekly just to make sure I am on track.”

Theme 2: Program supported patients' psychological health and well-being

**Provided participants with a sense of care**

- “I love the program. I’m single and live alone and it was nice to receive a message with helpful advice and it made me much less stressed, and I felt less alone.”
- “Was nice to get messages to know someone was caring and supportive through my new transitional time.”
- “The messages made me feel like there was someone checking on me and was steering me in the right direction.”
- “Great program. I felt that I had daily support/companion to manage my condition.”

**Participant accountability**

- “Keep it going. It helped me so much and made me feel that I was cared for, there was someone looking out for me and it kept me on my toes.”
- “It was a good program. It kept me informed of that I was supposed to do to stay healthy. A weekly reminder, at least, does not let you forget your obligations.”

**Motivation and positivity**

- “The messages were good motivation. It’s easy to revert away from a healthier lifestyle and forget about my condition but the but the messages kept it front of mind.”
- “I was glad to do it. It motivated me a lot. Kept on straight and narrow. I looked forward to receiving the massages. Thank you.”
- “The increase in psychological safety was a direct result. This helped in keeping me focused and in a positive state of mind as I journeyed through regaining my health and amending my lifestyle.”

### Implementation and Maintenance: Fidelity and Attrition

A total of 851,058 (average 97 per participant) SMS text messages were sent between April 21, 2020, and April 1, 2024; 99.41% (846,009/851,058) were successfully delivered. Of the 8804 patients who participated in the HeartHealth program, 9.5% (n=840) did not complete the 6-month program, 25.0% (210/840) withdrew from the program in the first week, 52.9% (444/840) withdrew between weeks 2 and 13, and 22.1% (186/840) withdrew between weeks 14 and 26. In comparison with those patients who completed the intervention, the group of participants who did not complete the 6-month intervention was younger (mean 56.89, SD 18.7 years vs mean 58.8, SD 15.8 years; *P*=.003), more likely to be female (42.5% vs 39.2%; *P*=.09), and had a lower prevalence of cardiovascular risk factors (hypertension 38.3% vs 48.9%, *P*<.001; hypercholesterolemia 35.2% vs 44%, *P*=.002; and diabetes mellitus 17.9% vs 24.3%, *P*<.001).

### Resources and Costs

The program was operationalized by 4 The University of Sydney—Westmead Applied Research Centre staff members on a part-time basis; these roles included program manager, digital product manager, health administrator, and research assistant, each with respective responsibilities as outlined in Multimedia Appendix 5. The resources and corresponding expenses required to operationalize the HeartHealth program over the 4 years were $276,728.36 (at the time of study analysis period completion [April 1, 2024], Aus $ to US $ was $0.65. Based on this, conversion to US $ is $179,873.43 [$20.43 per participant]). Since we initially provided this as an SMS text messaging–only recruitment strategy, and then later provided it as an SMS text messaging and phone call recruitment strategy, we have provided the separate estimated costs for each of these recruitment strategies in Multimedia Appendix 6. The estimated costs using the SMS text messaging–only recruitment strategy were $75,809.13 (at the time of study analysis period completion [April 1, 2024], Aus $ to US $ was $0.65. Based on this, conversion to US $ is $49,275.93 [$19.93 per participant]), and the estimated costs using the SMS text messaging and phone call recruitment strategy were $200,919.23 (at the time of study analysis period completion [April 1, 2024], Aus $ to US $ was $0.65. The cost converted to US $130,597.50 [$20.63 per participant]). A total of 2473 participants were recruited over the 18 months of the SMS text messaging–only recruitment period (137 per month), whereas a total of 6331 participants were recruited over the 30 months of the SMS text messaging and phone call period (211 per month).

### Participant SMS Text Message Interaction

Participants could reply to the messages or ask questions; it was not actively encouraged to reply, but program staff did monitor SMS text messages and respond as necessary. Across a total of 8804 people enrolled between April 2020 and April 2024, a total of 8288 responses were received. In total, 73% (6050/8804) of these responses were expressing thanks or acknowledging receipt of the message sent to them. The other 27% (2238/8288) of responses were based on lifestyle behaviors or administrative content.

### Barriers and Enablers to Implementing the HeartHealth Program

From a participant perspective, there were 2 main themes elicited as program enablers. First, the program content was valuable and appropriate, as it reinforced existing knowledge, improved cardiac health awareness, and improved engagement by providing relatable and actionable information. Second, the program was communicated in an effective manner, where the frequency of the messages provided steady reminders to participants and the URL hyperlinks allowed access to further information (Textbox 2). In contrast, the barriers identified by some participants were that information was not personalized enough, there were limited language options offered to participants, there was a lack of flexibility in the message delivery timing, and the content, at times, was overly simplistic (Multimedia Appendix 7).

### Adaptations Made When Implementing the HeartHealth Program

From a HeartHealth staff perspective, program feedback aligned with participant survey feedback on the perceived program use, the benefit of SMS text messaging personalization, and ease of program use. In addition, staff felt empowered and effective in their ability to manage and communicate with participants if issues arose (Multimedia Appendix 8). The main barriers perceived were lack of promotion of the program by local clinical staff, leading to participants being unaware of the program and the limited digital health literacy or English literacy of some participants, thereby requiring assistance from staff or family to onboard them to the program (Multimedia Appendix 9). Recommended future program adjustments were focused around overcoming these barriers, expanding program dissemination, and improving program personalization (Multimedia Appendix 10).

Three key adaptations were made by the HeartHealth staff following program commencement. First, efforts were made to increase “site staff program awareness” through emailing senior medical staff, presenting at departmental meetings, providing additional verbal and written education to all staff, and placing posters in wards, clinics, and frequented locations. Second, HeartHealth staff members made adaptations to “improve the enrollment process”; these adaptations aimed to improve patient understanding of the program and ease of enrollment through simplifying the initial enrollment SMS text message and implementing a follow-up phone call to assist in this process where required. In total, 19.1% (1390/7281) of the follow-up phone calls resulted in patients enrolling in the program during the call and 15.3% (1114/7281) enrolling following the call. Third, the SMS text message content was continually adapted throughout the program to provide up-to-date information on COVID-19, guidelines, and cardiovascular health (Multimedia Appendix 11).

Textbox 2. **Participant-reported enablers for implementing the HeartHealth program.**

Theme 1: Appropriate and valuable content was delivered

**SMS text messaging content reinforced existing knowledge**

- “Most of the information I knew but liked getting the messages to reinforce my knowledge.”
- “All the texts were very helpful and from memory, some were repeated which was fantastic as it kept reinforcing the message if I had not taken on board what the message was telling me.”

**SMS text messaging content provided awareness of their cardiac condition**

- “Messages were a reminder that despite feeling well I still have a chronic heart disease and need to take care about that.”
- **“**The message content wasn'’t the most important to me as was the reminder that I did need to consider my cardio health overall.”
- “The messages provided additional reminder/reinforcement of the need to pay regular attention to aspects of lifestyle that affect health and wellbeing particularly in the context of my medical conditions.”
- “For a brief moment every day I was reminded to do all I could for my health. My heart is of great concern to me and I have learnt a bit with the resources you have recommended.”

**Engaged with relatable information**

- “My favourite messages that explain a little about the science of heart conditions and how the text advice could assist with that.”
- “My favourite message was the one that described what happens to the body as a result of exercise in video format.”
- “Symptoms of a heart attack or related heart disease the viewer could have & what to do.
- “More points on how smoking alcohol and eating the wrong foods can damage your heart and body.”

**Engaged with actionable advice**

- “I liked the ideas, suggestions and reminders to get me thinking about what I could do more than the more-prescriptive messages.”
- “Diet messages about nuts and salad to avoid sugar and reduce cholesterol are very encouraging.”
- “They were all good, some better than others. I liked the ones with actionable [information].”

Theme 2: Information was communicated in an effective manner

**Frequency of SMS text messages serving as consistent reminders**

- “I have been to Weight Watchers many times and know what I should do to keep healthy but unless you are getting somebody or a text message every day , you go backwards. The daily reinforcement is the key to my success. I want to thank you all for allowing me to be a participant as you have certainly made a difference in keeping me healthy and happy.”
- “Extremely important educational information...However, the frequent texts reminders help to be mindful on following the diet, medication and exercise plan. Many thanks.”
- “The actual message was less important than the fact that they reminded me to be careful of diet and to exercise regularly.”

**Hyperlinks facilitated expanding knowledge**

- “My favourite messages were ‘healthy type of facts’ or ideas with hyperlinks. [This allowed] the recipient the option to investigate further.”
- “I was interested in the messages that provided links to more detailed and comprehensive advice, particularly about salt intake.”
- “In general the comments where helpful, however, some of the messages might have links for further help. E.g., There was a message which suggested using herbs to add flavour to reduce salt. Finding the relevant information was very hard to find. Actual suggestions or a link would have been a lot more helpful.”

## Discussion

### Principal Results

This paper describes the initial implementation and a detailed appraisal of an algorithm-driven personalized digital education and support program “HeartHealth” for patients with heart conditions. Key learnings of this study were that (1) the program was able to be implemented with high fidelity with relatively low-resource usage; (2) the majority of participants completed the 6-month program; however, program noncompletion was more commonly seen in patients of a younger age, female sex, and a lower prevalence of CVD risk factors; (3) most participants who completed the postintervention survey reported improved health and behavioral risk factors; (4) content analysis of feedback questionnaires indicated that program benefits were driven by improved self-efficacy, feeling psychologically supported, and initiating healthy lifestyle behaviors; and (5) further personalization and further engagement with local stakeholders could improve engagement and impact of the program.

The program had a high reach of the target population who were offered enrollment within a 4-year time frame. The high rates of enrollment were likely facilitated by the simple method of enrollment and that patients may have been motivated to address their heart health because of their recent hospitalization or clinic visit. It was notable that participants, compared with nonenrollee patients, were younger and, consistent with this, had fewer comorbidities. Recent studies that followed a similar program structure by automatically obtaining eligible patient contact details were also able to reach a large number of their targeted patients [21,22]. Opt-out models in cardiovascular rehabilitation programs have also shown significant increases in patients referred to the program compared with opt-in models [23,24]. Future adaptations to the HeartHealth program are required to optimize enrollment rates; using an opt-out model may be an effective option. Telephone detailing could increase enrollment and may be particularly helpful for older participants with more comorbidities. Our previous analyses have indicated that the HeartHealth program may be more effective in reducing hospitalization in older participants and could justify the additional resources for telephone detailing [19].

The content analysis of participants provides multiple insights into the reasons for improved health effects from the program. These factors included a combination of direct and indirect factors, such as the psychological or emotional support, continued light-touch connectivity, and increased self-efficacy that improved patient experiences and also encouraged positive behavior changes. These reasons align with prominent psychobehavioral theories such as the theory of planned behavior [25], social cognitive theory [26], and the self-determination theory [27], which should be further used when planning future program adaptations and dissemination. It is well recognized that providing emotional support is essential for patient-centered care [28,29] and when combined effectively with clinical care, it positively increases the patient experience [30-32]. Through the HeartHealth program providing an avenue of continual support via the SMS text messaging platform, participants were highly engaged with the program. Importantly, many replies were a general comment or to say “thanks,” rather than using the program as a modality to report health concerns requiring a reply from the health counselor, which is consistent with previous research [33]. This form of engagement provides patients with an increased sense of care and support without being resource-intensive and demanding on staff workload. To continue delivering an effective program, it is vital to consider both the education content and the emotional support provided by the program when considering program adaptations.

The HeartHealth program was implemented with high fidelity and low participant attrition rates. An important factor for the successful implementation was due to the simplicity of participation and wide acceptance of the program. The acceptance and usability of SMS text messaging programs have previously been demonstrated in other cohorts with CVD [11], as well as cohorts with mental health [34,35] and renal diseases [36] and diabetes [37]. Furthermore, HeartHealth staff reported that the successful implementation was partly enabled through an appropriate, easy-to-use program design, personalized patient contact upon program invitation, and skilled, adaptive staff members. For example, an adaptation initiated by staff members was to contact patients via a phone call to assist with enrollment if they had not responded to the SMS text messaging invitation, resulting in a large increase in enrollment. Previous studies have found self-enrollment to be a barrier for some patients, thereby highlighting the importance of providing assistance with enrollment or offering alternative enrollment modalities [38,39]. It is important to note that despite the HeartHealth program being implemented as a hospital service, the majority of the staff involved were from The University of Sydney. Lower engagement of local clinicians was identified as a barrier. This is consistent with previous observational studies of the implementation of new clinical services, finding that insufficient clinician time [40], lack of clinician motivation [41,42], high staff turnover [43], lack of continuing education [40,42], and an unsupportive organizational culture [40,41] were all barriers to implementation.

Participants identified that a lack of personalization of both message content and delivery was a main program barrier. A common theme from participants was that “one size does not fit all,” with the information not always being relevant and the message timing and modality not suiting all participants. The need for content personalization has been described previously in mHealth interventions among populations with CVD [44,45], and studies comparing personalized with nonpersonalized content on clinical outcomes in cardiovascular populations are lacking [46]; however, benefits have been shown in smoking populations [47]. Advances in machine learning and artificial intelligence will enable the development of personalization of content and responses to patient questions [48]; future research into the design and implementation evaluation of such programs is required.

To enable program sustainability and ongoing improvements, adaptations are required. HeartHealth staff and participants highlighted that the program could be further supported by incorporating a communication channel with health care members to provide information and support when required. It has been shown that patients may feel overwhelmed and unguided on where to find trusted information with the rising tide of health misinformation [49]. Patients with CVD have previously reported that they want their doctor or nurse to recommend information sources [50]. Future research should assess the feasibility and impact on clinical outcomes of an interactive SMS text messaging program before incorporating it into the HeartHealth program.

An important consideration with wider dissemination is the required cost and resources to implement the program. While a comprehensive cost-effectiveness analysis of the HeartHealth program is required, previous community SMS text messaging trials, using the TextCare platform, were found to be cost-saving and health improving in cohorts with CVD [51] and diabetes [52]. Other SMS text messaging programs have demonstrated program cost-effectiveness in patients with renal disease [53] and in smoking cessation campaigns [54]. The cost of the HeartHealth program was significantly less than that of traditional cardiac rehabilitation programs, which has previously been reported to cost between US $631 and US $1457 per participant, depending on the program setting (hospital, home, or remote) [55-57]. While the HeartHealth program is not designed as a replacement for traditional cardiac rehabilitation programs, it may serve as an effective adjunct. Overall, the HeartHealth program is likely to be appealing to other departments, given the frugal nature of the program, participant benefits, and low-resource requirements needed to implement the program.

### Limitations

There were limitations to this study. First, the postintervention survey had a moderate response rate (3533/7964, 44.4%); therefore, feedback may not be representative of all participants. This response rate is in keeping with previous online surveys and may be attributable to survey fatigue during and after the COVID-19 pandemic [58,59]. Second, we did not capture information from those participants who declined the initial SMS text messaging invitation, nor those who opted out of the program after enrollment; therefore, limiting our interpretation of the program’s reach and translational ability. Given the program having high participant acceptability and low-resource requirement to implement the program, evaluating long-term program maintenance, awareness, and funding sources will be pertinent for wider dissemination. Third, program effectiveness was assessed using patient-reported outcomes obtained only from a self-selected subgroup of program completers; therefore, the effectiveness results may overstate the true impact of the program due to this inherent bias. However, our previous research underpinning the HeartHealth program conducted using a randomized controlled design demonstrated improved lifestyle behaviors and cardiovascular risk factor profiles [11,16]. To assess program effectiveness more robustly, it would be beneficial to conduct a randomized controlled trial assessing the impact of the program on health care service usage. Fourth, as this was an evaluation of a program implemented into real-world clinical practice, we were limited in data collection and approval to extract linked medical record data. We were able to provide comorbidity data for HeartHealth participants and nonenrollee patients only for the period between April 2020 and April 2022. This limits the ability to describe and characterize those patients who opted in to the program and identify barriers to wider program adoption. Finally, the comorbidity data were drawn from both patient-reported information (“Fidelity and Attrition” section of the “Results” section) and linked medical record data diagnoses (Multimedia Appendix 4), with the latter potentially underrecording conditions such as hypercholesterolemia, hypertension, and diabetes mellitus, as these are typically diagnosed and managed in primary care settings. These differing data sources likely explain the lower prevalence discrepancy of hypercholesterolemia, hypertension, and diabetes mellitus in the “Results” section and in Multimedia Appendix 4.

### Implications

The adoption of mobile health technologies has significantly risen over recent years, which can largely be attributed to the COVID-19 pandemic, a heightened focus on telehealth, and the increasing burden on health care services [60]. SMS text messaging programs within cardiovascular populations have been shown to be an effective modality to improve cardiovascular health; however, to date, there have been few that have proceeded to large-scale implementation and a formal scientific evaluation. This study demonstrates the feasibility and use of implementing personalized postdischarge support at low cost. It also identifies important enablers and barriers to implementation. Future scale-up should consider further customization of programs to individuals and broadening availability through personalized language and health literacy.

### Conclusions

This thorough implementation evaluation highlights the successful implementation of the HeartHealth program. Participant attrition and perceived lifestyle benefits demonstrate the program effectiveness; additionally, staff and participant feedback has highlighted key program barriers and enablers. These insights provide key learnings for future scale-up and improvement of HeartHealth postdischarge digital support.

## Supplementary material

10.2196/68896 Multimedia Appendix 1 Examples of text messages used in the HeartHealth program.

10.2196/68896 Multimedia Appendix 2 HeartHealth postintervention participant survey.

10.2196/68896 Multimedia Appendix 3 HeartHealth staff focus group discussion topics.

10.2196/68896 Multimedia Appendix 4 HeartHealth participant and nonenrollee participant cardiovascular comorbidities and English as preferred language data between April 2020 and April 2022 (adapted with permission from Sheahen et al [19]).

10.2196/68896 Multimedia Appendix 5 Roles and duties of HeartHealth staff.

10.2196/68896 Multimedia Appendix 6 Costs associated with the HeartHealth program over the 4-year analysis period, with breakdown of costs during the initial text messaging–only recruitment period during the first 18 months of the program (April 2020 to October 2021) and the subsequent text messaging and phone call recruitment period used during the following 30 months (October 2021 to April 2024).

10.2196/68896 Multimedia Appendix 7 Participant barriers for implementing the HeartHealth program.

10.2196/68896 Multimedia Appendix 8 HeartHealth staff enablers for implementing the HeartHealth program.

10.2196/68896 Multimedia Appendix 9 HeartHealth staff barriers for implementing the HeartHealth program.

10.2196/68896 Multimedia Appendix 10 HeartHealth staff recommended future adaptations for implementing the HeartHealth program.

10.2196/68896 Multimedia Appendix 11 Organization adaptations made to implementing the Heart Health program.

## Abbreviations

CVD: cardiovascular disease
REDCap: Research Electronic Data Capture

## References

[1] (2023). Therapeutic patient education: an introductory guide. World Health Organization.

[2] (2023). Improving patient education: a new guide for policy-makers and health professionals to support self-management of chronic conditions. World Health Organization.

[3] Warsi A, Wang PS, LaValley MP, Avorn J, Solomon DH (2004). Self-management education programs in chronic disease: a systematic review and methodological critique of the literature. Arch Intern Med.

[4] (2012). Reducing risk in heart disease: an expert guide to clinical practice for secondary prevention of coronary heart disease. Cardiac Society of Australia and New Zealand.

[5] Kwan T, Chua B, Pires D, Feng O, Edmiston N, Longman J (2022). A qualitative analysis of the barriers and enablers faced by Australian rural general practitioners in the non-pharmacological management of congestive heart failure in community dwelling patients. BMC Health Serv Res.

[6] Dhaliwal KK, King-Shier K, Manns BJ, Hemmelgarn BR, Stone JA, Campbell DJT (2017). Exploring the impact of financial barriers on secondary prevention of heart disease. BMC Cardiovasc Disord.

[7] Sérvio TC, Britto RR, de Melo Ghisi GL (2019). Barriers to cardiac rehabilitation delivery in a low-resource setting from the perspective of healthcare administrators, rehabilitation providers, and cardiac patients. BMC Health Serv Res.

[8] Kinsman L, Tham R, Symons J, Jones M, Campbell S, Allenby A (2016). Prevention of cardiovascular disease in rural Australian primary care: an exploratory study of the perspectives of clinicians and high-risk men. Aust J Prim Health.

[9] Rogers HL, Pablo Hernando S, Núñez-Fernández S (2021). Barriers and facilitators in the implementation of an evidence-based health promotion intervention in a primary care setting: a qualitative study. J Health Organ Manag.

[10] (2024). Mobile services and coverage. Department of Infrastructure, Transport, Regional Development, Communications, Sport and the Arts.

[11] Chow CK, Redfern J, Hillis GS (2015). Effect of lifestyle-focused text messaging on risk factor modification in patients with coronary heart disease: a randomized clinical trial. JAMA.

[12] Tam HL, Wong EML, Cheung K, Chung SF (2021). Effectiveness of text messaging interventions on blood pressure control among patients with hypertension: systematic review of randomized controlled trials. JMIR Mhealth Uhealth.

[13] Skinner R, Gonet V, Currie S, Hoddinott P, Dombrowski SU (2020). A systematic review with meta-analyses of text message-delivered behaviour change interventions for weight loss and weight loss maintenance. Obes Rev.

[14] Whittaker R, McRobbie H, Bullen C, Rodgers A, Gu Y (2016). Mobile phone-based interventions for smoking cessation. Cochrane Database Syst Rev.

[15] Ross ES, Sakakibara BM, Mackay MH (2021). The use of SMS text messaging to improve the hospital-to-community transition in patients with acute coronary syndrome (Txt2Prevent): results from a pilot randomized controlled trial. JMIR Mhealth Uhealth.

[16] Klimis H, Thiagalingam A, McIntyre D, Marschner S, Von Huben A, Chow CK (2021). Text messages for primary prevention of cardiovascular disease: the TextMe2 randomized clinical trial. Am Heart J.

[17] Redfern J, Thiagalingam A, Jan S (2014). Development of a set of mobile phone text messages designed for prevention of recurrent cardiovascular events. Eur J Prev Cardiol.

[18] Klimis H, Thiagalingam A, Chow CK (2020). Text messages for primary prevention of cardiovascular disease: the TextMe2 randomised controlled trial protocol. BMJ Open.

[19] Sheahen B, Marschner S, Min H (2025). Implementation and impact of postdischarge support of cardiovascular patients using text messages the HeartHealth program. JACC Adv.

[20] Drisko JW, Maschi T (2016). Content Analysis.

[21] Bressman E, Long JA, Honig K (2022). Evaluation of an automated text message-based program to reduce use of acute health care resources after hospital discharge. JAMA Netw Open.

[22] Clarke M (2023). Impact of an automated text and phone call postdischarge follow-up program on patient satisfaction scores and 30-day hospital readmission among adult patients. https://hsrc.himmelfarb.gwu.edu/son_dnp/123.

[23] Adusumalli S, Jolly EG, Chokshi NP, Washington LA, Suchoicki L, Walsh MM (2017). Abstract 19699: a change in cardiac rehabilitation referral defaults from opt-in to opt-out increases referral rates among patients with ischemic heart disease. Circulation.

[24] Adusumalli S, Jolly E, Chokshi NP (2021). Referral rates for cardiac rehabilitation among eligible inpatients after implementation of a default opt-out decision pathway in the electronic medical record. JAMA Netw Open.

[25] Etheridge C, Sinyard RD, Brindle ME, Osband AJ, Newell PC, Bakal JA, Eltorai AEM (2023). Translational Surgery.

[26] Bandura A (2001). Social cognitive theory: an agentic perspective. Annu Rev Psychol.

[27] Mayo NL, Russell HA, Holt K, Williams GC (2022). Implementation of a self-determination based clinical program to reduce cardiovascular disease risk. J Health Psychol.

[28] Tzelepis F, Sanson-Fisher RW, Zucca AC, Fradgley EA (2015). Measuring the quality of patient-centered care: why patient-reported measures are critical to reliable assessment. Patient Prefer Adherence.

[29] Bradshaw J, Siddiqui N, Greenfield D, Sharma A (2022). Kindness, listening, and connection: patient and clinician key requirements for emotional support in chronic and complex care. J Patient Exp.

[30] Han E, Shiraz F, Haldane V (2019). Biopsychosocial experiences and coping strategies of elderly ESRD patients: a qualitative study to inform the development of more holistic and person-centred health services in Singapore. BMC Public Health.

[31] Allen D, Scarinci N, Hickson L (2018). The nature of patient- and family-centred care for young adults living with chronic disease and their family members: a systematic review. Int J Integr Care.

[32] Rathert C, Williams ES, McCaughey D, Ishqaidef G (2015). Patient perceptions of patient-centred care: empirical test of a theoretical model. Health Expect.

[33] Singleton AC, Raeside R, Hyun KK (2023). A National Health and Wellness SMS text message program for breast cancer survivors during COVID-19 (EMPOWER-SMS COVID-19): mixed methods evaluation using the RE-AIM framework. J Med Internet Res.

[34] Patel S, Akhtar A, Malins S (2020). The acceptability and usability of digital health interventions for adults with depression, anxiety, and somatoform disorders: qualitative systematic review and meta-synthesis. J Med Internet Res.

[35] Shariful Islam SM, Chow CK, Redfern J (2019). Effect of TEXT messaging on depression in patients with coronary heart disease: a substudy analysis from the TEXT ME randomised controlled trial. BMJ Open.

[36] Dawson J, Campbell KL, Craig JC (2021). A text messaging intervention for dietary behaviors for people receiving maintenance hemodialysis: a feasibility study of KIDNEYTEXT. Am J Kidney Dis.

[37] Middleton T, Constantino M, McGill M (2021). An enhanced SMS text message-based support and reminder program for young adults with type 2 diabetes (TEXT2U): randomized controlled trial. J Med Internet Res.

[38] Speirs KE, Grutzmacher SK, Munger AL, Messina LA (2016). Recruitment and retention in an SMS-based health education program: lessons learned from Text2BHealthy. Health Informatics J.

[39] Gazmararian JA, Elon L, Yang B, Graham M, Parker R (2014). Text4baby program: an opportunity to reach underserved pregnant and postpartum women?. Matern Child Health J.

[40] Wallis L (2012). Barriers to implementing evidence-based practice remain high for U.S. nurses. Am J Nurs.

[41] Ramón C, Nievas-Soriano BJ, García-González J, Alarcón-Rodríguez R, Requena-Mullor M, Lozano-Paniagua D (2022). Motivation and barriers to research among nursing professionals in Southeast Spain. Healthcare (Basel).

[42] Curtis K, Fry M, Shaban RZ, Considine J (2017). Translating research findings to clinical nursing practice. J Clin Nurs.

[43] Baron AN, Hemler JR, Sweeney SM (2020). Effects of practice turnover on primary care quality improvement Implementation. Am J Med Qual.

[44] Atluri N, Mishra SR, Anderson T (2024). Acceptability of a text message‐based mobile health intervention to promote physical activity in cardiac rehabilitation enrollees: a qualitative substudy of participant perspectives. J Am Heart Assoc.

[45] Coorey GM, Neubeck L, Mulley J, Redfern J (2018). Effectiveness, acceptability and usefulness of mobile applications for cardiovascular disease self-management: systematic review with meta-synthesis of quantitative and qualitative data. Eur J Prev Cardiolog.

[46] Shariful Islam SM, Farmer AJ, Bobrow K (2019). Mobile phone text-messaging interventions aimed to prevent cardiovascular diseases (Text2PreventCVD): systematic review and individual patient data meta-analysis. Open Heart.

[47] Lin H, Li M, Xiao L, Chang C, Liu GG (2023). Efficacy of personalised text message intervention in reducing smoking frequency and amount for non-abstinent smokers: a double-blind, randomised controlled trial. J Glob Health.

[48] Farmer A, French D, Bartlett K, Klonoff DC, Kerr D, Espinoza JC (2024). Diabetes Digital Health, Telehealth, and Artificial Intelligence.

[49] Daraz L, Morrow AS, Ponce OJ (2019). Can patients trust online health information? A meta-narrative systematic review addressing the quality of health information on the internet. J Gen Intern Med.

[50] Wells S, Mahony F, Lee A (2024). Preferred format and strategies for seeking and trusting online health information: a survey of cardiology outpatient attendees across three New Zealand hospitals. J Prim Health Care.

[51] Burn E, Nghiem S, Jan S (2017). Cost-effectiveness of a text message programme for the prevention of recurrent cardiovascular events. Heart.

[52] Islam SMS, Peiffer R, Chow CK (2020). Cost-effectiveness of a mobile-phone text messaging intervention on type 2 diabetes—a randomized-controlled trial. Health Policy Technol.

[53] Dawson J, Howell M, Howard K (2022). Cost-effectiveness of a mobile phone text messaging program (KIDNEYTEXT) targeting dietary behaviours in people receiving haemodialysis. J Hum Nutr Diet.

[54] Cobos-Campos R, Mar J, Apiñaniz A (2021). Cost-effectiveness analysis of text messaging to support health advice for smoking cessation. Cost Eff Resour Alloc.

[55] Edwards K, Jones N, Newton J (2017). The cost-effectiveness of exercise-based cardiac rehabilitation: a systematic review of the characteristics and methodological quality of published literature. Health Econ Rev.

[56] Oehler AC, Holmstrand EC, Zhou L (2024). Cost analysis of remote cardiac rehabilitation compared with facility-based cardiac rehabilitation for coronary artery disease. Am J Cardiol.

[57] Southard BH, Southard DR, Nuckolls J (2003). Clinical trial of an Internet-based case management system for secondary prevention of heart disease. J Cardiopulm Rehabil.

[58] de Koning R, Egiz A, Kotecha J (2021). Survey fatigue during the COVID-19 pandemic: an analysis of neurosurgery survey response rates. Front Surg.

[59] Krieger N, LeBlanc M, Waterman PD, Reisner SL, Testa C, Chen JT (2023). Decreasing survey response rates in the time of COVID-19: implications for analyses of population health and health inequities. Am J Public Health.

[60] (2023). Admitted patient care. Australian Institute of Health and Welfare.

