# Supplementary material for: The HeartHealth Program: A Mixed Methods Study of a Community-Based Text Messaging Support Program for Patients With Cardiovascular Disease From 2020 to 2024
Source: JMIR Cardio. 2026 Mar 11;10:e68896. doi: 10.2196/68896 (PMC12978537; doi:10.2196/68896)
Supplement: Multimedia Appendix 1 [file cardio-v10-e68896-s001.docx]

**Multimedia Appendix 1**

Examples of SMS messages used in the HeartHealth program.

| **Smoking** | |
| --- | --- |
|  | [NAME], when quitting smoking – enlist your Dr’s help, try a nicotine chewing gum, patches or inhaler. |
|  | Hi [NAME] smoking increases the risk of heart disease by up to 6 times. To learn more visit [URL] |
| **Diet** | |
|  | Hi NAME], did you know the goal for an average adult is to consume less than 1 teaspoon of salt per day? To learn more visit [URL] |
|  | Hi [NAME try leaving a plate of chopped fruit on the table to graze on after dinner. |
| **Physical activity** | |
|  | Have you exercised this week [NAME]? We recommend at least 30mins of moderate-intensity activity a day. If 30mins is too much at once, do 10 min at a time, until you reach 30. |
|  | Hi [NAME], activity can be accumulated in shorter bouts of 10 minutes each. Look at these tips: [URL link] |
| **COVID-19** | |
|  | More people are working from home or isolating themselves and social isolation is associated with heart unhealthy behaviors. It’s good to be aware of this, try to talk with others every day and try and keep active. |
|  | COVID19 vaccination reduces the risk of being hospitalised. Read here for useful info on how being vaccinated protects you. [URL] |
| **General cardiovascular information** | |
|  | It’s hard to remember to take tablets every day! Try & make it easier – put them next to your toothbrush or somewhere easy to remember. |
|  | Do you know the warning signs of heart attack [NAME]? To learn more visit [URL] |
