# Supplementary material for: The HeartHealth Program: A Mixed Methods Study of a Community-Based Text Messaging Support Program for Patients With Cardiovascular Disease From 2020 to 2024
Source: JMIR Cardio. 2026 Mar 11;10:e68896. doi: 10.2196/68896 (PMC12978537; doi:10.2196/68896)
Supplement: Multimedia Appendix 2 [file cardio-v10-e68896-s002.docx]

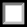


Multimedia Appendix 2

Thank you for your participation in the Text Care Research Study. We seek your feedback about the program so we can improve it in the future. All responses are strictly confidential.

| **PART A – Feedback about the program** | | | | | |
| --- | --- | --- | --- | --- | --- |
| *Please read each statement below and tick one option for each.* | | | | | |
| **a) As a result of the messages, my diet became more healthy** | | | | | |
| 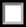 | 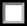 |  | 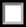 | 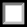 | 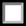 |
| Strongly agree | Agree |  | Neutral | Disagree | Strongly disagree |
| **e) As a result of the messages, I increased my exercise (physical activity) levels** | | | | | |
| 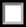 | 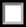 |  | 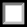 | 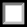 | 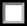 |
| Strongly agree | Agree |  | Neutral | Disagree | Strongly disagree |
| **f) The messages helped remind me to take my medicines** | | | | | |
| 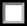 | 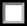 |  | 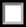 | 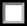 | 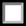 |
| Strongly agree | Agree |  | Neutral | Disagree | Strongly disagree |

| **PART B - Additional information** |
| --- |
| *Please provide any further information relevant to the following questions* |
| **i) What did you find most useful? What was your favourite message(s)?** |
| **ii) What did you find least useful? Were there message(s) you didn’t like?** |
| **iii) Do you have any suggestions to improve our program?** |
| **PART D – General comments** |
|  |

Thank you very much for your participation. Your input to the development of this health education program is very valuable.
