# Supplementary material for: The HeartHealth Program: A Mixed Methods Study of a Community-Based Text Messaging Support Program for Patients With Cardiovascular Disease From 2020 to 2024
Source: JMIR Cardio. 2026 Mar 11;10:e68896. doi: 10.2196/68896 (PMC12978537; doi:10.2196/68896)
Supplement: Multimedia Appendix 3 [file cardio-v10-e68896-s003.docx]

**Multimedia Appendix 3**

| **Part A: Occupation description** |
| --- |
| **Please provide a description of your role in HeartHealth** |
| **Part B: Fidelity** |
| **In the beginning, how was the HH program intended to be implemented?**  Prompts:   - - Aims   - On-boarding   - Maintenance   - Technology   - Staff |
| **Did the program get implemented as planned?**  Prompts:   - - No – What changes were made?   - Yes – What allowed the program to be implemented as planned? |
| **What adaptations were made to improve the program implementation?**  Prompts:   - - Patient enrollment / on-boarding   - Staff   - Technology |
| **Part C: Barriers / Enablers / Adaptations** |
| **What factors have allowed the program to be effectively implemented?**  Prompts:   - - Organisational factors   - Individual factors   - Technology factors   - Leadership/Team factors |
| **Are there any factors which have prevented the program from being optimally implemented?** |
| **What costs are associated with implementing the HeartHealth program** |
| **Part D: Future Dissemination** |
| **What may be the barriers to implementing HeartHealth in other sites / departments?** |
| **What would be required to allow the HeartHealth program to be implemented at more sites throughout the state/country?** |
