# Supplementary material for: The HeartHealth Program: A Mixed Methods Study of a Community-Based Text Messaging Support Program for Patients With Cardiovascular Disease From 2020 to 2024
Source: JMIR Cardio. 2026 Mar 11;10:e68896. doi: 10.2196/68896 (PMC12978537; doi:10.2196/68896)
Supplement: Multimedia Appendix 4 [file cardio-v10-e68896-s004.docx]

**Multimedia Appendix 4**

| **Characteristic** | **Control**,  N = 7218  Mean (SD); n / N (%) | **Intervention**,  N = 4324  Mean (SD); n / N (%) | **Overall**,  N = 11542  Mean (SD); n / N (%) |
| --- | --- | --- | --- |
| **English as preferred language** | 5109/6722 (76.0%) | 3268/3979 (82.1%) | 8377/10701 (78.3%) |
| Missing | 496 | 345 | 841 |
| **Hypertension** | 1001/7218 (13.7%) | 422/4324 (9.8%) | 1423/11542 (12.3%) |
| **Hypercholesterolaemia** | 81/7218 (1.1%) | 34/4324 (0.8%) | 115/11542 (1%) |
| **Diabetes mellitus** | 1100/7218 (15.4%) | 471/4324 (10.9%) | 1581/11542 (13.7%) |
| **Obesity** | 582/7218 (8.1%) | 256/4324 (5.9%) | 838/11542 (7.3%) |
| **Alcohol misuse disorder** | 63/7218 (0.9%) | 23/4324 (0.5%) | 86/11542 (0.74%) |
| **Smoker** | 1429/7218 (19.8%) | 599/4324 (13.8%) | 2028/11542 (17.6%) |
| **Heart failure** | 2362/7218 (32.7%) | 1099/4324 (25.4%) | 3461/11542 (30%) |
| **Ischaemic heart disease** | 1441/7218 (20%) | 656/4324 (15.2%) | 2097/11542 (18.2%) |
| **Acute Coronary Disease** | 1045/7218 (14.5%) | 470/4324 (10.9%) | 1515/11542 (13.13%) |
| **Atrioventricular block** | 285/7218 (3.9%) | 109/4324 (2.5%) | 394/11542 (3.4%) |
| **Atrial fibrillation** | 1176/7218 (16.3%) | 538/4324 (12.4%) | 1714/11542 (14.8%) |
| **Supraventricular tachycardia** | 223/7218 (3.1%) | 175/4324 (4.1%) | 398/11542 (3.4%) |
| **Ventricular tachycardia and fibrillation** | 339/7218 (4.7%) | 178/4324 (4.1%) | 517/11542 (4.5%) |
| **Sick sinus syndrome** | 149/7218 (2.1%) | 67/4324 (1.6%) | 216/11542 (1.9%) |
| **Cardiac arrest** | 82/7218 (1.1%) | 37/4324 (0.9%) | 119/11542 (1%) |
|  | | | |
