# Supplementary material for: The HeartHealth Program: A Mixed Methods Study of a Community-Based Text Messaging Support Program for Patients With Cardiovascular Disease From 2020 to 2024
Source: JMIR Cardio. 2026 Mar 11;10:e68896. doi: 10.2196/68896 (PMC12978537; doi:10.2196/68896)
Supplement: Multimedia Appendix 5 [file cardio-v10-e68896-s005.docx]

**Multimedia Appendix 5**

| **Role** | **Duties** |
| --- | --- |
| Program manager | 1. Maintaining relationship with WSLHD 2. Maintaining relationship with cardiology departments 3. Compliance with ethics and governance |
| Digital product manager | 1. Budgeting, quotes, and payments 2. Addressing TextCare issues |
| Health administrator | 1. Uploading participant details to the TextCare platform 2. Monitoring and archiving participant details 3. Monitoring and responding to participant messages 4. Fortnightly and monthly reports of program use |
| Research assistant | 1. Follow-up enrollment phone calls 2. Follow-up contact to participant concerns/queries |
