# Supplementary material for: The HeartHealth Program: A Mixed Methods Study of a Community-Based Text Messaging Support Program for Patients With Cardiovascular Disease From 2020 to 2024
Source: JMIR Cardio. 2026 Mar 11;10:e68896. doi: 10.2196/68896 (PMC12978537; doi:10.2196/68896)
Supplement: Multimedia Appendix 6 [file cardio-v10-e68896-s006.docx]

**Multimedia Appendix 6**

| **Roles** | **Cost** |
| --- | --- |
| **Once only fee** | |
| Program set up | $600 |
| **Fixed infrastructure/software fees** | |
| System maintenance | $1,000 per month |
| Virtual mobile number | $6.5 per month |
| **Variable infrastructure fee** | |
| Messages | **Outgoing: 6c per message**  Total 4-year period cost: $51,063.48  Cost during SMS-only recruitment period: $14,348.84  Cost during SMS & Phone call recruitment period: $36,714.64  **Incoming: 1c per message**  Total 4-year period cost: $82.88  Cost during SMS-only recruitment: $23.29  Cost during SMS & Phone call recruitment: $59.59 |
| **Variable personnel fee** |  |
| Monitoring and Follow-up phone calls: Research assistant role | Total 4-year period cost: $57,850  Cost during SMS-only recruitment ($75 per week): $5850  Cost during SMS & Phone call recruitment ($400 per week): $52,000 |
| Technical support: Health administrator and Digital support manager roles | Total 4-year period cost: $113,100  Cost during SMS-only recruitment ($450 per week): $35,100  Cost during SMS & Phone call recruitment ($600 per week): $78,000 |
| Program set-up and initial implementation: Program manager role | Total 4-year period cost; however, the program manager role was only required for the initial 24-month period ($55 per week): $5,720 |
| **Overall cost of 4-year HeartHealth analysis period** | Total 4-year period cost: $276,728.36 ($31.43 per participant)  SMS-only recruitment: $75,809.13 ($30.65 per participant)  SMS & Phone call recruitment: $200,919.23 ($31.74 per participant) |
| * Total participants recruited in 4-year period: 8804. Estimated number of participants recruited in the SMS-only recruitment period of 18 months (April 2020 to October 2021): 2473. Estimated number of participants recruited in the SMS & Phone call recruitment period of 30 months (October 2021 to April 2024): 6331. | |
