# Supplementary material for: The HeartHealth Program: A Mixed Methods Study of a Community-Based Text Messaging Support Program for Patients With Cardiovascular Disease From 2020 to 2024
Source: JMIR Cardio. 2026 Mar 11;10:e68896. doi: 10.2196/68896 (PMC12978537; doi:10.2196/68896)
Supplement: Multimedia Appendix 7 [file cardio-v10-e68896-s007.docx]

**Multimedia Appendix 7.**

Participant barriers for implementing the HeartHealth Program.

| **Theme 1: Message content personalisation** |
| --- |
| **Information was too basic** |
| “Many of the messages were what I consider common knowledge and common sense” |
|  |
| “Too many messages were for basic diet and exercise information, things I already knew from the cardiac rehab program and from information I had gained from doctors and dietitians already.” |
|  |
| “Thinking about it I think the wording, there seemed to be a presumption that you didn't know about the information provided and sometimes patronising. [I recommend] casual, engaging wording that takes into consideration there is a broad range of knowledge amongst participants and not everyone is eating unhealthy food and not exercising. |
|  |
| **Information was not relevant to all patients** |
| “I am an unusual patient as I am very fit and healthy for my age.  The messages were just not relevant to my lifestyle as I was already doing the majority of things suggested.” |
|  |
| “Some messages weren't appropriate for my other medical conditions whilst I appreciate the message them I couldn't follow them.” |
|  |
| “A large number of messages were not relevant to my condition,  so that led to my being less careful, over time, with reading the messages.” |
|  |
| “Seems like this program has lumped all people with a heart related issue into one bucket.  It would be better if it was more tailored to the particular issue the patient has.” |
|  |
| **Theme 2: Message delivery personalisation** |
| **Timing of delivery** |
| “Messages arrived at all hours of the day. It would be better if they arrived post working hours (for those who are working).” |
|  |
| **Modality of delivery** |
| “I would rather get messages on my computer. I would easily be able to save messages and refer back to them. The text is too small and difficult to read at my age.” |
|  |
| “Not being tech savvy, emails would have been great so that I could have accessed the links on the [internet] via my computer, rather than my phone.” |
|  |
| “If it was an app with app pushed messages then it could remain longer or even indefinitely as a constant reminder to motivate patients.” |
|  |
| **Language customisation** |
| “Maybe in other languages for the elderly whom English is not their first language might struggle with comprehension.” |
