# Supplementary material for: The HeartHealth Program: A Mixed Methods Study of a Community-Based Text Messaging Support Program for Patients With Cardiovascular Disease From 2020 to 2024
Source: JMIR Cardio. 2026 Mar 11;10:e68896. doi: 10.2196/68896 (PMC12978537; doi:10.2196/68896)
Supplement: Multimedia Appendix 8 [file cardio-v10-e68896-s008.docx]

**Multimedia Appendix 8**

| **Enablers for implementing Heart Health Program** |
| --- |
| **Theme 1: Participant personalisation** |
| **Addressing participant by preferred name** |
| “We call like from their first name so that makes them like very familiar and they think that somebody is looking after them… so they’re feeling very close to the team, especially during COVID.” |
|  |
| **Incorporation of healthcare team** |
| **“**I have a column of clinicians who referred them [participants] and I often use that clinician to say this clinician thought this may be of benefit to you, And once they hear that name they sort of feel OK.” |
|  |
| **Theme 2: Appropriate program design** |
| **Simplicity of intervention** |
| *“*It’s just simple. Something that you know…anyone can interact with. You don't need any complicated apps or network or anything… you don't even need a smartphone.” |
|  |
| **Implementation timing** |
| *“*I think COVID play a big part… It was just the timing. I mean, we would have done it and it would've taken a little bit of time, but it was just the right timing I think as well. |
|  |
| “It was during COVID so the approval process happened very quickly - correct me if I'm wrong - because they wanted patients to get support and without having to come to the hospital. So we got quick approval, the break was approved and we enrolled in the programme very quickly. That’s been quite smooth” |
|  |
| **Ease of use** |
| “I think it’s also the fact that like you said [Participant 1], they don’t have to come to the hospital. So initially when I call and say this is it, they’re like oh I don't time to come in. I say oh well this is a bit like a friend sending you a text message. You can access it anytime you're free.” |
|  |
| **Program duration** |
| “From that evidence [User survey] mentioned, six months was just the right amount. When you go above… the satisfaction starts to decline. People are not as happy. When you go less people say always we want more” |
|  |
| **Theme 3: Effective program team members** |
| **Participant communication** |
| *“*Our patient facing… is very good at recruiting and very good on the phone to talk to patients, put them at ease. It makes a huge difference.” |
|  |
| **Escalating participant concerns** |
| “[Health administrator] is very quick at responding when we get something to be escalated, so you know, messages don't stay there for weeks before somebody can do something about it… We have a 72-hour time frame for us to answer to them, but we never wait until then, we answer within 24 hours always.” |
|  |
