# Supplementary material for: The HeartHealth Program: A Mixed Methods Study of a Community-Based Text Messaging Support Program for Patients With Cardiovascular Disease From 2020 to 2024
Source: JMIR Cardio. 2026 Mar 11;10:e68896. doi: 10.2196/68896 (PMC12978537; doi:10.2196/68896)
Supplement: Multimedia Appendix 9 [file cardio-v10-e68896-s009.docx]

**Multimedia Appendix 9**

HeartHealth staff barriers for implementing the HeartHealth Program.

| **Barriers for implementing Heart Health Program** |
| --- |
| **Theme 1: Site engagement** |
| **High staff turnover leading to poor engagement** |
| “I think the issue is more so engagement to making sure that the cardiology team is, you know, stay connected. There’s changeover of staff all the time. New interns, new residents come, how do we make sure that information flows from one to the next? In that sense I think it’s been challenging.” |
|  |
| **Site awareness of program information** |
| “The nurses, a patient could ask them a question, or the junior doctors. So I think we need awareness at some level. Even don’t need to know details, but just some awareness what it is, and who to contact. And because our challenge is none of us are based in the clinic. And that makes it harder.” |
|  |
| “The issue we had is [The doctors] changing and when they change they won’t pass [Program information] to the new team. So we had an issue there” |
|  |
| **Theme 2: Participant capacity and concerns** |
| ***Digital literacy*** |
| “The verification code is very difficult for the elderly to be able to get a verification code, go on another screen and then put in the answer. I know when I started it, and I did it, it took me three goes to do it.” |
|  |
| **Privacy fears** |
| “They just want to know whether – first of all whether it’s a hoax or not, that’s the main thing.” |
|  |
| **Non English-speaking background** |
| “The consent message, the first message which goes to these participants on Monday so they may sometimes misunderstood because most of them are… maybe non-English speaking background.” |
