# Supplementary material for: The HeartHealth Program: A Mixed Methods Study of a Community-Based Text Messaging Support Program for Patients With Cardiovascular Disease From 2020 to 2024
Source: JMIR Cardio. 2026 Mar 11;10:e68896. doi: 10.2196/68896 (PMC12978537; doi:10.2196/68896)
Supplement: Multimedia Appendix 10 [file cardio-v10-e68896-s010.docx]

**Multimedia Appendix 10**

| **Theme 1: Need for greater site staff involvement** |
| --- |
| **Increased program awareness** |
| “[Administrative] staff to nurses, to the junior doctors, to consultants. All of them, because our admin team are the ones that receive the call about Heart Health, and if they [site stakeholders] don’t know it, they would just say this is nothing to do with the clinic for example. So they need to know. The nurses, a patient could ask them a question, or the junior doctors. So I think we need awareness at some level” |
|  |
| “It's literally just conversing I guess and making sure that there is awareness. In a form of common transparency. So if it's here’s at our quarterly report, showing the outcomes just in an email, or if it is a presentation to the staff, you know or like [Health administrator] mentioned the posters, we put new posters up, look at the beautiful design, something like that.” |
|  |
| ‘Writing discharge summaries as a junior doctor, they always put a plan and that's always at the top. Maybe it's something talking to the junior doctors and saying, as your last point in your plan it should say, this [Heart Health program] is something you will be contacted about, it's been very beneficial so far so keep your ear out for an invite.” |
|  |
| **Demonstrate the program success to the cardiology team** |
| “We're doing great stuff and we're trying to show them everything that's happening and the outcomes of what we do and how the participants actually feel about it… It's like a pat on the back that you know this is going great, everyone’s happy. This is the percentage of people that actually enrolled, this is the amount that said that they actually enjoying this maybe we should push it a bit more” |
|  |
| **Theme 2: Further program personalisation** |
| **Monitoring engagement with SMS messages** |
| “But also from our side, being able to look at what's happening so at the moment if someone doesn't get a message we are aware of that but have they actually opened it? Have they properly interacted with that [Message]” |
|  |
| **Establish a protocol to personalise SMS messages** |
| “It's a matter of the workflow… Because yes, we can have it all done manually where any of us sits down and decides on [Relevant messages for participants] but obviously that's [going to] be a massive increase in workload to do that for hundreds of patients… But then again if you have some evidence or a consistent flow…. we can straightaway apply and go from there and maybe adjust if needed even more.” |
|  |
| **Improve participant convenience** |
| “I think we are at a good position as it is now, but there is always room for improvement and I think making it that one step more convenient for the participants. Even if it's simple as shortening the links or simple as just reminders, stuff like that. Something that would easily make them more interact with the actual content that's available there. |
|  |
| **Theme 3: Wider program dissemination** |
| **Government funding** |
| *“*LHDs [Local Health Districts] might support as well. So, if we collect enough information and what you’re doing is going to be reporting that this has been beneficial and we have achieved, you know, patients love it etc., then we should be able to get LHDs to fund it as a support programme.” |
|  |
| **Increased staffing** |
| “We're [going to] need a bigger team. That's definitely first. Specifically, [Research assistant and health administrator] roles. |
|  |
| **Ethics approval and ensuring patient privacy** |
| “If we’re going to be accessing patient details from other hospitals so that, we need proper ethical approvals and making sure that we’re all on the same page. Otherwise, confidentiality becomes an issue” |
|  |
| **Improved enrollment workflow** |
| “I would see the onboarding would be a bit difficult… If we’re managing both or multiple hospitals or sectors. So I mean ultimate optimisation for that would be great…. Something to keep in mind if we do want to move for the future instead of having to do it manually, it’d be great. |
