# Supplementary material for: The HeartHealth Program: A Mixed Methods Study of a Community-Based Text Messaging Support Program for Patients With Cardiovascular Disease From 2020 to 2024
Source: JMIR Cardio. 2026 Mar 11;10:e68896. doi: 10.2196/68896 (PMC12978537; doi:10.2196/68896)
Supplement: Multimedia Appendix 11 [file cardio-v10-e68896-s011.docx]

**Multimedia Appendix 11**

| **Adaptations made for implementing Heart Health Program** |
| --- |
| **Theme 1: Site member program awareness** |
| **Implementation of program posters** |
| *“*We were successful actually with the posters… In the right clinic or the wards as well as all the clinic rooms, the individual rooms, yeah. We have a contact number here to contact us…. So they call me and ask about the programme, so I explain it to them.” |
|  |
| **Site member education** |
| “We give them a leaflet detailing description, a summary. So then they are aware if somebody comes and asks them, they are aware about the programme and they will tell and they will give my contact details for them to contact me.” |
|  |
| **Presenting at department meetings** |
| “We have tried a few things. A while ago there used to be regular meetings for cardiology, so we try to present at those meetings since to see if they can, you know people stay informed.” |
|  |
| **Theme 2: Improved participant enrollment process** |
| **Follow-up enrollment phone calls** |
| “We thought that some patients may require assistance, you know someone to guide them through, give them that ease that, you know this is OK, this is genuine and also if they need some technical support of which you know how to register. So that’s why we trialled with [Phone calls] and realised that the rate of registration involvement increased.” |
|  |
| **Simplifying the initial enrollment message** |
| “The consent message, the first message which goes to these participants on Monday so they may sometimes misunderstood because most of them are… maybe non-English speaking background as well. So then actually that time I think [Heart Health team], we made it simpler, especially [Heart Health cardiologists] made that text message text very simple and after that actually, we don’t have that issue anymore.” |
|  |
